# Quantitative Contrast-Enhanced Ultrasound Characterization of Perilesional Perfusion and Its Association with CD34-Positive Cell Density in a Rat Model of Hepatic Alveolar Echinococcosis

**DOI:** 10.3390/pathogens15090987

**Published:** 2026-09-17

**Authors:** Huan Zhao, Ya Xu, Xinyao Li, Zhen Wang, Yuhang Cheng, Jingyu Wang, Changhui Qu, Dan Dong, Ting Ma, Jian Dong

**Affiliations:** 1 Department of Ultrasound, First Affiliated Hospital of Shihezi University, Shihezi 832008, China; 2 School of Medicine, Shihezi University, Shihezi 832003, China

**Keywords:** hepatic alveolar echinococcosis, contrast-enhanced ultrasound, time–intensity curve, CD34-positive cell density, microvascular perfusion, perilesional zone

## Abstract

Hepatic alveolar echinococcosis (HAE) is characterized by infiltrative growth and remodeling of perilesional host tissue, including microvascular changes. We investigated whether quantitative contrast-enhanced ultrasound (CEUS) could characterize perilesional perfusion and its animal-level association with CD34-positive cell density in a standardized rat model. Twenty female Sprague Dawley rats underwent ultrasound-guided intrahepatic HAE modeling and CEUS 10 weeks later. Peak enhancement (PE), rise time (RT), time to peak (TTP), wash-in rate (WiR), wash-in perfusion index (WiPI), and wash-in area under the curve (WiAUC) were measured in the perilesional zone and reference liver parenchyma. CD34-positive cell density was quantified by digital pathology. The perilesional zone showed higher PE, WiR, WiPI, and WiAUC and shorter RT and TTP than the reference liver parenchyma (all FDR-adjusted *p* < 0.001). CD34-positive cell density correlated positively with PE (ρ = 0.696, FDR-adjusted *p* = 0.004), WiR (ρ = 0.525, FDR-adjusted *p* = 0.026), WiPI (ρ = 0.614, FDR-adjusted *p* = 0.012), and WiAUC (ρ = 0.534, FDR-adjusted *p* = 0.026), but not with RT or TTP. Within this experimental model, these findings indicate increased and accelerated perilesional perfusion and support quantitative CEUS as a contrast-enhanced imaging-based approach for characterizing its relationship with local microvascularity.

## 1. Introduction

Hepatic alveolar echinococcosis (HAE), caused by *Echinococcus multilocularis*, is characterized by pseudotumoral, infiltrative growth within the liver and may progressively involve surrounding hepatic tissue and vascular structures [1,2,3]. Because of this infiltrative growth pattern and the accompanying host tissue response, the lesion–liver interface is particularly relevant to understanding local disease activity and host–parasite interactions [1,4]. Histopathologically, this interface is characterized by inflammatory and fibrotic reactions around parasitic structures, while vascular remodeling may also occur in the perilesional tissue [4,5].

Microvascular remodeling is an important component of this perilesional response. Experimental studies have shown that angiogenic signaling contributes to HAE progression and that inhibition of vascular endothelial growth factor-related pathways can suppress parasite-associated lesion growth [5]. Histologically, CD34 immunohistochemistry is commonly used to label endothelial structures and assess tissue microvascularity, and automated or digital quantification of CD34-stained tissue has been used to provide continuous vascular measurements [6,7]. These findings provide a pathological basis for evaluating whether alterations in local blood perfusion at the HAE margin can be detected with contrast-enhanced imaging.

Routine ultrasonography, computed tomography, and magnetic resonance imaging provide complementary information on HAE lesion morphology and extent [8,9]. CEUS enables real-time assessment of microbubble transit, with TIC analysis providing quantitative measurements [10,11]. Previous clinical and experimental studies have described peripheral enhancement in HAE and its association with histological microvascularity [12,13,14,15,16,17,18,19,20]. Quantitative and semiquantitative CEUS evaluation has therefore already been introduced into HAE research. Building on that evidence, the present study applied a paired regional, multiparametric TIC workflow to relate six perfusion measurements to continuous animal-level CD34-positive cell density. The contribution is the characterization of parameter-specific associations, supported by measurement reproducibility and sensitivity analyses, rather than the discovery of a vascularized rim. This framework may inform the selection of candidate perfusion measures for subsequent longitudinal studies; the present study did not test diagnostic superiority or clinical outcomes.

## 2. Materials and Methods

### 2.1. Experimental Animals

Twenty female specific pathogen-free Sprague Dawley (SD) rats, aged 6–8 weeks and weighing 200–220 g, were obtained from SiPaiFu Biotechnology Co., Ltd. (Beijing, China). Published evidence has demonstrated the feasibility of ultrasound-guided hepatic inoculation, with successful model establishment and survival of all animals in the reported experimental cohort [21]. Sample-size adequacy for the correlation analysis was assessed using a Fisher *z*-based approximation. Assuming a correlation coefficient of r = 0.60, a two-sided α = 0.05, and 80% statistical power, the estimated requirement was 19.34 analyzable animals, rounded up to 20. Animals were housed at the Laboratory Animal Center of Shihezi University School of Medicine under controlled conditions of 22 ± 2 °C, a 12 h light–dark cycle, and 50–60% relative humidity, with free access to standard rodent chow and sterile filtered water. The rats were acclimatized for 7 days before the experiment. All procedures were approved by the Bioethics Committee of Shihezi University before study initiation (A2024-291).

### 2.2. Isolation of E. multilocularis Protoscoleces and Establishment of the Rat Model

Under aseptic conditions, *E. multilocularis* metacestode tissue was harvested from infected Mongolian gerbils and finely minced. The tissue was suspended in ice-cold phosphate-buffered saline (PBS) containing 1% penicillin–streptomycin, mechanically dispersed, passed through a 100 µm cell strainer, and washed with prechilled PBS. Protoscolex viability was assessed by eosin exclusion and was approximately 85%. Based on the viable protoscolex count, the suspension was diluted to a final concentration of approximately 4.0 × 10^4^ viable protoscoleces/mL.

**Figure 1:**
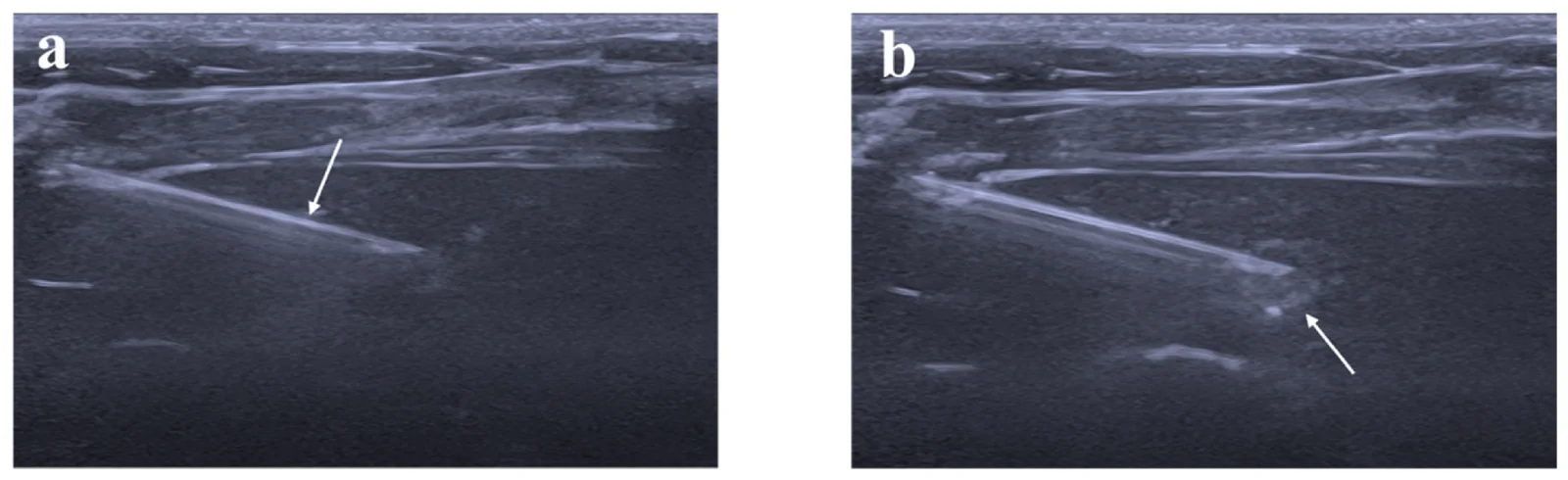
Ultrasound-guided percutaneous intrahepatic inoculation in SD rats. Note: (**a**) Needle placement in the left lateral hepatic lobe; the arrow indicates the needle tract. (**b**) Injection of a suspension of *E. multilocularis* protoscoleces; the arrow indicates the hyperechoic injection site.

For both modeling and imaging, anesthesia was induced with 5% isoflurane in oxygen for 2–3 min and maintained with 1.5% isoflurane delivered continuously through a nose cone. A warming pad set to 37 °C was used, and the respiratory rate was monitored throughout. Using an ultrasound-guided percutaneous intrahepatic inoculation approach adapted from a previously reported method [21], 0.05 mL of suspension containing approximately 2000 viable protoscoleces was injected into the left lateral hepatic lobe of each rat under ultrasound guidance (Figure 1). Ultrasound examinations were performed 10 weeks after inoculation under the same anesthetic and temperature control protocol.

### 2.3. Conventional Ultrasound Examination

Conventional ultrasound examinations were performed using a Mindray Resona A20T system (Mindray Bio-Medical Electronics Co., Ltd., Shenzhen, China) equipped with an L33-8U linear transducer (8.0–33.0 MHz). Rats were fasted for at least 6 h before scanning.

The liver was examined systematically to determine lesion number, and the maximum lesion diameter was measured on the largest cross-sectional plane. When more than one hepatic lesion was present, the largest lesion was designated as the target lesion for subsequent CEUS and histopathological analysis.

### 2.4. Contrast-Enhanced Ultrasound Examination

CEUS was performed after conventional ultrasound using a Samsung RS85 system (Samsung Medison, Seoul, Republic of Korea) equipped with an LA2-14A linear transducer (2.0–14.0 MHz). To ensure the comparability of quantitative measurements, the same ultrasound system, transducer, contrast-specific preset, and acquisition settings were used for all animals and remained unchanged throughout image acquisition. Imaging was performed at a low mechanical index of 0.08 to minimize microbubble disruption.

SonoVue (Bracco, Milan, Italy) was reconstituted with 10 mL of 0.9% saline. A 0.4 mL bolus was administered through a preimplanted tail-vein catheter, followed immediately by a 1.0 mL saline flush. Dynamic acquisition was initiated simultaneously with contrast administration. The transducer position and imaging plane were kept as stable as possible throughout acquisition. Continuous cine loops were recorded for 120 s and exported in the Digital Imaging and Communications in Medicine (DICOM) format.

The 120 s acquisition period was sufficient to capture contrast arrival, wash-in, and peak enhancement for TIC analysis. Washout-related parameters were not included because the study was designed to characterize wash-in and peak-related perfusion features.

### 2.5. Image Analysis

Two senior ultrasound physicians, each with more than 10 years of clinical ultrasound experience, independently analyzed the stored CEUS cine loops. Both observers were blinded to each other’s measurements and to the histopathological results.

Quantitative perfusion analysis was performed using VueBox software (version 7.7; Bracco Suisse SA, Plan-les-Ouates, Switzerland). The perilesional zone was defined as the rim-like hyperenhancing tissue surrounding the persistently nonenhancing lesion center. A reference frame showing the clearest peripheral enhancement, usually at or near peak enhancement, was selected for ROI placement.

**Figure 2:**
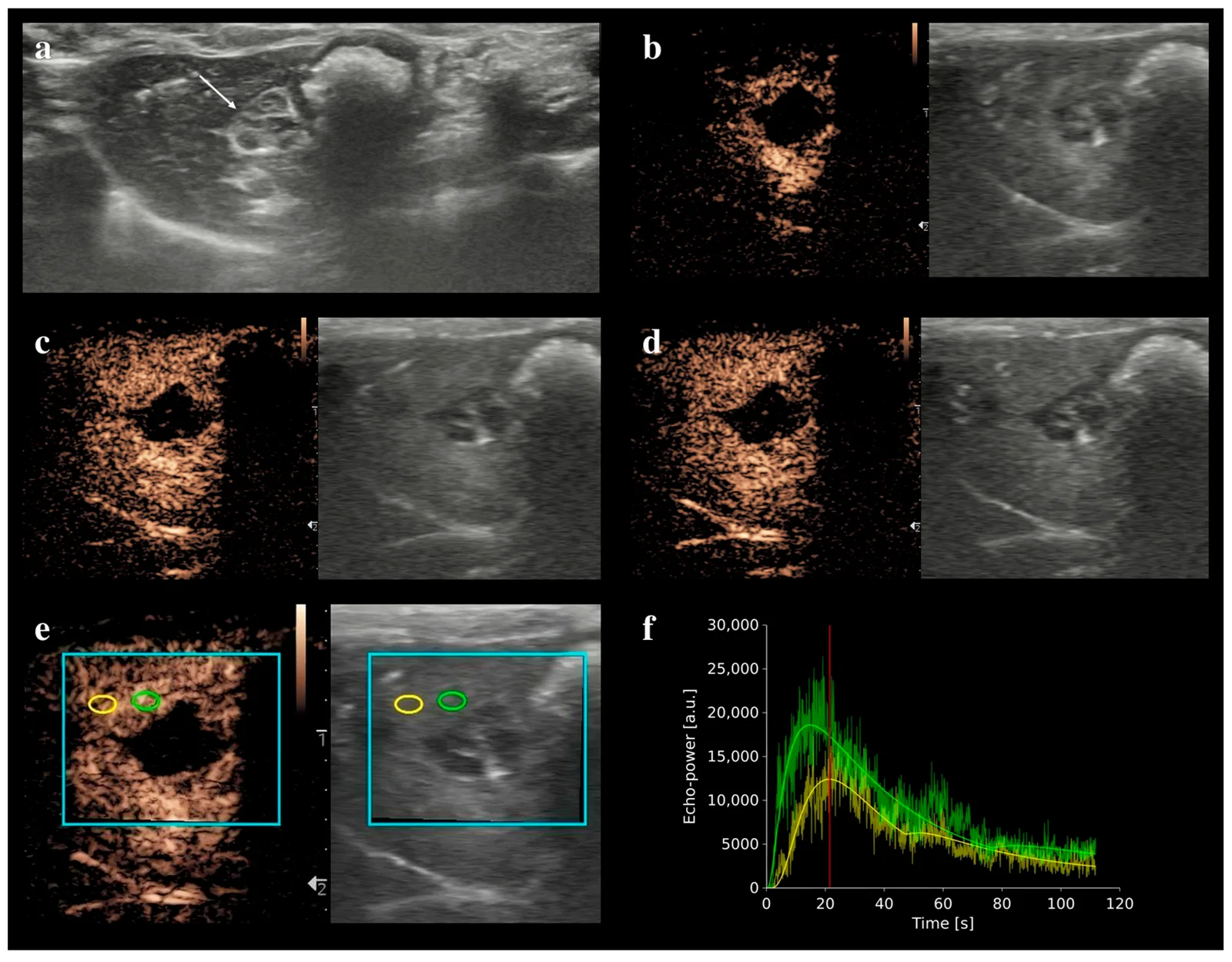
CEUS images and time–intensity curves of an HAE lesion and reference liver parenchyma. Note: (**a**) Two-dimensional ultrasound appearance of HAE in an SD rat. (**b**–**d**) Representative frames from the 120 s CEUS cine loop during early wash-in, peak enhancement, and a later acquisition point. (**e**,**f**) TIC analysis of the same lesion: the perilesional ROI is shown in green and the reference liver ROI in yellow.

A manual region of interest (ROI) was placed within a relatively homogeneous portion of the perilesional zone, avoiding large vessels, cystic or nonenhancing areas, and imaging artifacts. The ROI area was adapted to the extent of the selected enhancing tissue and was not fixed across animals. For each rat, a reference ROI of the same area was placed in a visually unremarkable liver parenchyma outside the perilesional zone at a similar imaging depth, while similarly avoiding large vessels and artifacts. The software’s respiratory motion compensation function was used to track both ROIs throughout the cine loop, with manual correction when necessary (Figure 2).

Both observers independently delineated the ROIs and completed quantitative CEUS measurements for all 20 animals according to the same predefined criteria. The mean of their first measurements was used for regional comparisons and imaging–pathology association analyses.

Four weeks after the initial analysis, Observer 1 repeated the quantitative CEUS measurements for all animals, after the case order had been randomized and without access to the original ROIs or measurements. The first measurements from Observers 1 and 2 were used to assess interobserver reproducibility, whereas the first and repeat measurements from Observer 1 were used to assess intraobserver reproducibility.

Six TIC-derived parameters were recorded: peak enhancement (PE, arbitrary units [a.u.]), rise time (RT, s), time to peak (TTP, s), wash-in rate (WiR, a.u.), wash-in perfusion index (WiPI, a.u.), and wash-in area under the curve (WiAUC, a.u.).

### 2.6. Histopathological Analysis

Immediately after CEUS, the rats were euthanized with an overdose of isoflurane. The probe position and acoustic beam direction corresponding to the largest cross-sectional plane of the target lesion were marked after imaging and before laparotomy. During tissue collection, the corresponding lesion was identified and cut along the recorded beam direction through its largest plane, then oriented for embedding to approximate the CEUS plane. Tissue was fixed in 4% paraformaldehyde for 48 h, embedded in paraffin, and sectioned at 4 µm for H&E staining and CD34 immunohistochemistry.

H&E staining was performed according to standard procedures. For immunohistochemistry, sections underwent citrate buffer antigen retrieval, endogenous peroxidase blockade, and serum blocking. Sections were then incubated with anti-CD34 antibody (1:150; Boster Biological Technology, Wuhan, China; BM4082), followed by secondary antibody incubation, DAB development, and hematoxylin counterstaining.

Whole-slide images were acquired using an LG-S80 scanner and analyzed on the Saiviewer platform with AIpathwell digital pathology software (version 1.2; Servicebio Technology Co., Ltd., Wuhan, China). One experienced pathologist, blinded to the CEUS measurements, first identified the perilesional inflammatory infiltration zone according to tissue morphology and then selected 10 fields randomly within that zone for each rat at 40× digital magnification. Within each selected field, an ROI was delineated along the morphological boundaries of the inflammatory infiltration tissue. The ROI shape and area were adapted to the extent of this tissue within the field, without imposing a fixed ROI area. CD34-positive cell density was calculated by dividing the number of CD34-positive cells within each ROI by its actual analyzed tissue area and was expressed in cells/mm^2^.

The mean of the CD34-positive cell densities from the 10 fields was used as the pathological measurement for each rat. Because this endpoint quantified CD34-positive cells per unit tissue area rather than discrete vascular profiles, it was treated as a digital measure of local microvascularity rather than as conventional microvessel density.

### 2.7. Statistical Analysis

Statistical analyses were performed using SPSS version 27.0 (IBM Corp., Armonk, NY, USA) and Python 3.13 with SciPy 1.17.0. Continuous variables are presented as the mean ± standard deviation or median (interquartile range), as appropriate.

For comparisons between the perilesional zone and reference liver parenchyma, the normality of the within-animal paired differences was assessed using the Shapiro–Wilk test. Parameters with paired differences compatible with a normal distribution were analyzed using paired-samples *t*-tests, whereas those with non-normally distributed paired differences were analyzed using two-sided exact Wilcoxon signed-rank tests. For paired-samples *t*-tests, mean paired differences and *t*-based 95% confidence intervals (CIs) were reported. For Wilcoxon signed-rank tests, median within-animal differences and bias-corrected and accelerated (BCa) 95% CIs based on 50,000 animal-level bootstrap resamples were reported as descriptive effect estimates. Differences were defined as perilesional zone minus reference liver values.

Associations between CEUS parameters and CD34-positive cell density were assessed using Spearman’s rank correlation as the prespecified primary analysis. Correlation CIs were estimated using 50,000 BCa animal-level bootstrap resamples. As sensitivity analyses, CD34 labels were permuted across rats 100,000 times to obtain two-sided Monte Carlo *p*-values using a plus-one correction, and leave-one-out analysis omitted each rat once and recalculated all six correlations and their within-iteration Benjamini–Hochberg adjustment in the remaining 19 animals. The six regional comparisons and the six CEUS–pathology correlations were treated as separate hypothesis families and adjusted using the Benjamini–Hochberg FDR procedure. Exploratory associations between the maximum lesion diameter and each CEUS parameter were also evaluated by Spearman’s correlation, with FDR adjustment across the six size–parameter tests.

Interobserver reproducibility was evaluated using two-way random-effects, absolute-agreement intraclass correlation coefficients for single measurements [ICC(A,1)] and for the mean of two observers [ICC(A,2)]. Intraobserver reproducibility for Observer 1 was assessed using a two-way mixed-effects, absolute-agreement ICC(A,1) [22]. Ninety-five percent CIs were estimated from 10,000 paired animal-level bootstrap resamples. ICC point estimates were interpreted as poor (<0.50), moderate (0.50 to <0.75), good (0.75–0.90), or excellent (>0.90), while also considering CI precision [22]. For PE, WiR, WiPI, and WiAUC, Bland–Altman analyses were performed separately for both ROI types and for interobserver (Q1 − Q2) and intraobserver (Q1 − Q1-W4) comparisons. Bias and 95% limits of agreement (LoAs) were calculated as the mean paired difference ± 1.96 × SD of paired differences. The standard error of measurement (SEM) was reported in native units, with SEM% used for cross-parameter descriptive comparison. Because no a priori clinically acceptable error boundary was defined, LoA and SEM were interpreted descriptively.

All tests were two-sided, and an FDR-adjusted *p* < 0.05 was considered statistically significant.

## 3. Results

All 20 rats developed hepatic lesions and were included in the final analysis, with no deaths or exclusions. At 10 weeks after inoculation, mean body weight was 299.14 ± 20.19 g. The mean maximum lesion diameter was 9.73 ± 3.82 mm, with a range of 3.9–15.5 mm. Fourteen rats (70.0%) had a single hepatic lesion, and six (30.0%) had multiple lesions (≥2). Maximum lesion diameter was not significantly correlated with PE, RT, TTP, WiR, WiPI, or WiAUC (all FDR-adjusted *p* = 0.910; Supplementary Table S2).

All target lesions showed persistent central nonenhancement with a discernible peripheral hyperenhancing rim during wash-in and peak enhancement, and all TICs reached peak enhancement within the 120 s acquisition period. Across the 20 rats, the CEUS ROI area was 1.34 ± 0.26 mm^2^ (range, 0.90–1.90 mm^2^), with equal-sized ROIs used for the perilesional zone and reference liver parenchyma within each rat.

After FDR correction, all six TIC parameters differed between the perilesional zone and reference liver parenchyma. PE was higher in the perilesional zone (28,197.36 ± 7030.72 vs. 16,314.34 ± 3077.40 a.u.; mean paired difference, 11,883.02; 95% CI, 8697.01–15,069.02), as were WiR [4069.44 (2744.70–5120.19) vs. 1357.95 (1065.44–1603.97) a.u.; median paired difference, 2467.08; 95% CI, 1689.78–3234.99], WiPI [20,229.57 (16,286.73–22,889.84) vs. 8284.60 (7733.29–9891.50) a.u.; median paired difference, 10,180.54; 95% CI, 8153.88–13,403.12], and WiAUC (263,018.63 ± 89,548.90 vs 172,188.46 ± 41,883.36 a.u.; median paired difference, 62,348.61; 95% CI, 37,006.40–125,441.99). RT was shorter (13.01 ± 3.48 vs. 19.82 ± 4.37 s; mean paired difference, −6.81; 95% CI, −8.76 to −4.85), as was TTP (16.26 ± 4.25 vs. 21.94 ± 5.28 s; median paired difference, −4.59; 95% CI, −7.04 to −3.13). All regional comparisons had FDR-adjusted *p* < 0.001 (Table 1).

**Figure 3:**
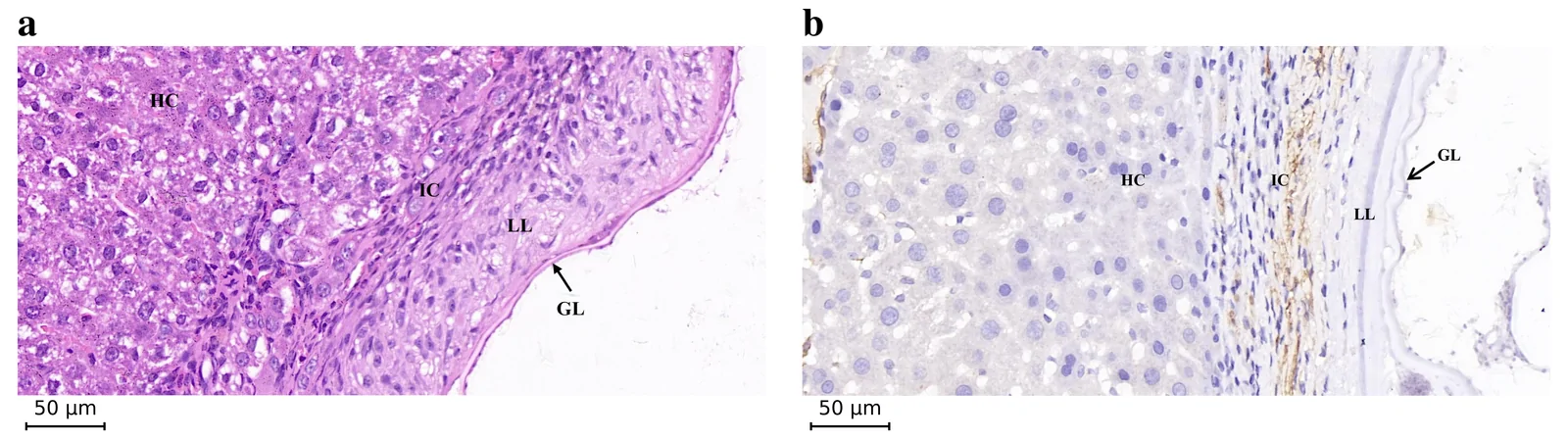
Histopathological and immunohistochemical features of the perilesional zone. Note: (**a**) H&E staining shows the inflammatory interface surrounding the parasitic lesion. Nuclei appear blue-purple, whereas eosin-stained tissue appears pink. (**b**) CD34 immunohistochemistry shows CD34-positive structures in the perilesional zone. Brown staining indicates CD34 immunoreactivity, and nuclei are counterstained blue with hematoxylin. Arrows indicate the germinal layer. Digital magnification, 40×; scale bars, 50 μm. HC, hepatocyte; IC, inflammatory cell; GL, germinal layer; LL, laminated layer.

H&E staining showed granulomatous inflammation and fibrosis around the parasitic structures, with laminated and germinal layers visible within the lesion (Figure 3a). CD34-positive cells and vascular-associated structures were prominent at the perilesional interface (Figure 3b). The mean histological ROI area per rat (averaged over 10 fields) was 0.04121 ± 0.03591 mm^2^, with a range of 0.00437–0.14843 mm^2^ across the 20 rats. The mean CD34-positive cell density was 2680.53 ± 365.67 cells/mm^2^.

**Figure 4:**
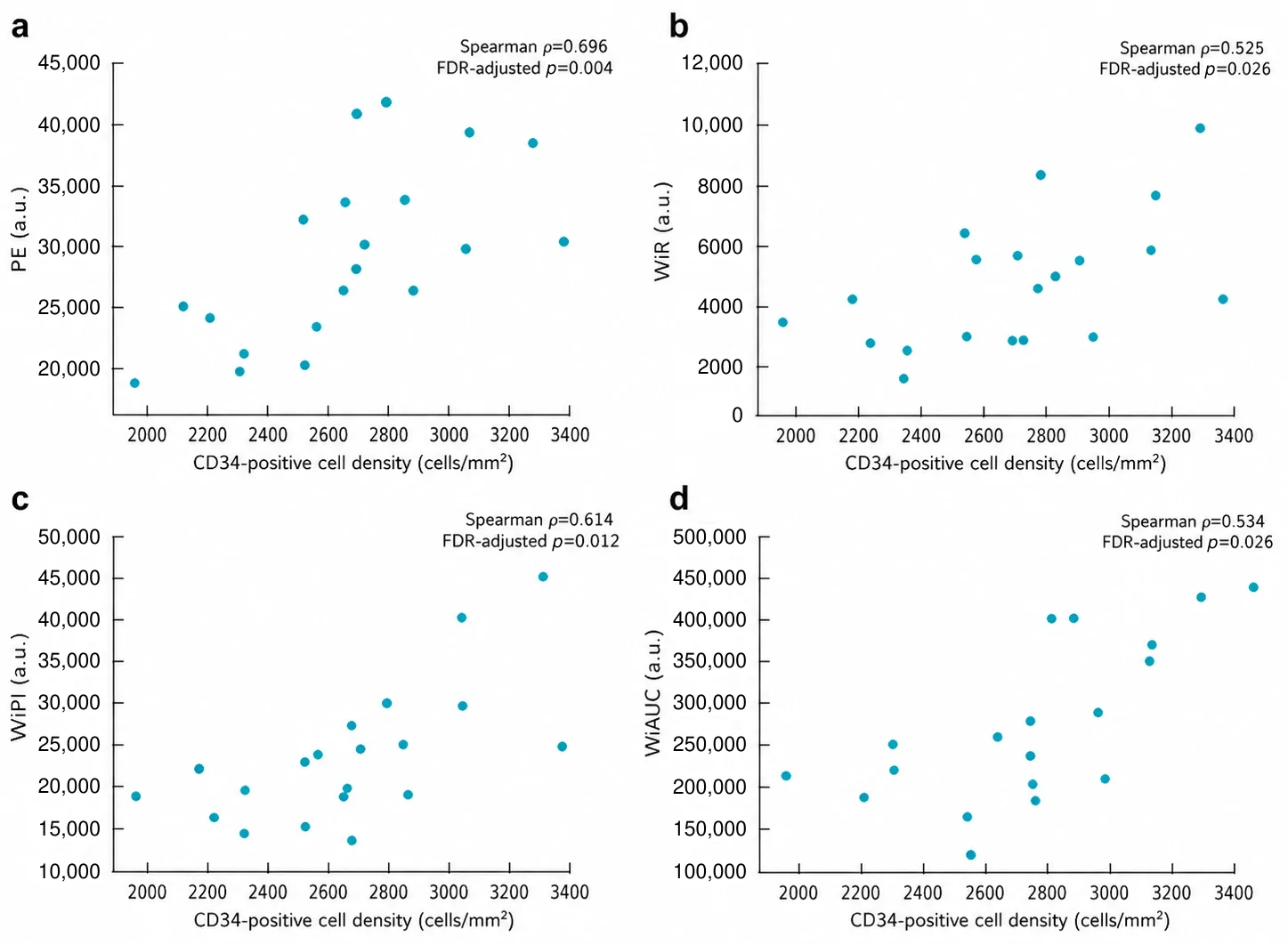
Associations of PE, WiR, WiPI, and WiAUC with CD34-positive cell density (cells/mm^2^). Note: (**a**) PE versus CD34-positive cell density; (**b**) WiR versus CD34-positive cell density; (**c**) WiPI versus CD34-positive cell density; (**d**) WiAUC versus CD34-positive cell density. Each point represents one rat (n = 20). CEUS values are the animal-level means of the two observers’ initial measurements used in the primary analysis. Spearman’s ρ and FDR-adjusted *p*-values are shown.

After FDR correction, CD34-positive cell density was positively correlated with PE (ρ = 0.696, FDR-adjusted *p* = 0.004), WiR (ρ = 0.525, FDR-adjusted *p* = 0.026), WiPI (ρ = 0.614, FDR-adjusted *p* = 0.012), and WiAUC (ρ = 0.534, FDR-adjusted *p* = 0.026), whereas no significant correlations were observed for RT or TTP (Table 2; Figure 4). BCa CIs, permutation sensitivity, and leave-one-out results supported the direction of the four positive associations, although the WiR and WiAUC leave-one-out significance counts were less uniform than those for PE and WiPI (Supplementary Table S1).

Complete first and repeat measurements were available for all 20 animals. Across the 12 parameter-by-ROI combinations, interobserver ICC(A,1) values ranged from 0.759 to 0.873, interobserver ICC(A,2) values ranged from 0.863 to 0.932, and intraobserver ICC(A,1) values ranged from 0.801 to 0.932 (Supplementary Table S3). The widest CI was observed for perilesional WiPI [interobserver ICC(A,1) = 0.759, 95% CI 0.256–0.894]. In the 16 Bland–Altman analyses of PE, WiR, WiPI, and WiAUC, 18–20 of 20 paired observations (90–100%) were within the 95% LoA. The interobserver SEM% ranged from 8.10% to 21.09%, and the intraobserver SEM% ranged from 6.63% to 24.17% (Supplementary Tables S4 and S5; Supplementary Figure S1).

## 4. Discussion

In this rat model of HAE, multiparametric TIC analysis demonstrated a distinct perilesional perfusion pattern compared with a reference liver parenchyma. PE, WiR, WiPI, and WiAUC were higher, whereas RT and TTP were shorter. PE, WiR, WiPI, and WiAUC were positively associated with CD34-positive cell density, whereas no significant associations were observed for RT or TTP. Collectively, these findings support enhancement magnitude and wash-in measures, particularly PE, WiR, WiPI, and WiAUC, as promising quantitative CEUS markers for characterizing perilesional perfusion and CD34-positive microvascularity in experimental HAE. Further studies directly comparing the correlation coefficients are warranted to determine their relative performance.

A recent clinical study classified HAE lesions according to CEUS enhancement phenotypes and examined their associations with pathological characteristics and vascular invasion [20], while experimental studies have also suggested a relationship between peripheral enhancement and histological microvascularity [15,16,17]. Notably, all target lesions in the present study showed peripheral rim enhancement with central nonenhancement, whereas a broader spectrum of CEUS phenotypes has been reported in clinical HAE [18,19,20]. This relative uniformity may partly reflect the standardized experimental setting. All animals underwent the same modeling procedure and were examined at the same time point, 10 weeks after inoculation, which may have reduced variability in the lesion stage and host background. By contrast, clinical HAE encompasses lesions at different stages and with more heterogeneous pathological and vascular features [18,19,20]. Building on these observations, the present study treated the perilesional zone as a distinct region of analysis, used multiparametric TIC analysis to quantify regional perfusion, and further assessed the associations between individual perfusion parameters and animal-level CD34-positive cell density. TIC-derived parameters capture different aspects of enhancement magnitude, wash-in kinetics, cumulative perfusion, and temporal behavior [10,11], allowing the microcirculatory characteristics of the perilesional zone to be described from multiple dimensions. Histopathologically, HAE lesions are surrounded by inflammatory and fibrotic host responses [4,14], while experimental studies have demonstrated increased microvascularity within the peripheral infiltration and proliferation zone [16,17]. The lesion center, by contrast, commonly shows absent or limited contrast enhancement [12,13,14,15,16,17]. Previous rat studies have linked the degree of peripheral CEUS enhancement to histological microvascularity [16,17]. The paired quantitative comparison between the perilesional zone and reference liver parenchyma in the present study further supports the interpretation that rim enhancement reflects a characteristic local hemodynamic feature of the infiltrative interface.

Clinical HAE encompasses heterogeneous sonographic appearances, MRI morphology, anatomical extent, host responses, and treatment histories [3,18,19,20,23]. These dimensions should not be conflated: a hailstorm appearance is a conventional ultrasound morphology descriptor, Kodama classification describes MRI morphology, and WHO-IWGE PNM staging describes disease extent. The uniform rim enhancement pattern in this experiment may partly reflect standardized inoculation and assessment at a single 10-week time point. Quantitative perilesional CEUS may complement morphological imaging by providing continuous perfusion information at the lesion–liver interface, with potential applications in monitoring change and selecting regions for further evaluation. Whether it identifies viable parasite-containing tissue, treatment response, or a parasite-free resection margin requires longitudinal human studies with matched pathology and outcomes. The contribution of this paper is a standardized quantitative description of the perilesional interface rather than rediscovery of rim enhancement. Six TIC-derived measures were linked to a continuous pathological endpoint in a paired regional design, with uncertainty, influence, and reproducibility assessment. The observed association pattern provides a basis for selecting candidate perfusion measures for future longitudinal studies. The current cross-sectional experiment does not, however, establish prediction of activity, progression or treatment response.

Further examination of the relationships between individual TIC parameters and CD34-positive cell density showed that regional perfusion differences did not necessarily parallel their pathological associations. PE reflects peak enhancement magnitude, WiR describes the rate of signal increase during contrast wash-in, WiPI integrates perfusion information during the wash-in phase, and WiAUC represents cumulative enhancement over this period [10,11]. All four parameters were positively correlated with CD34-positive cell density in the present study. This finding suggests that a richer microvascular network at the perilesional interface may provide a vascular basis for greater microbubble delivery and distribution, resulting in an increased enhancement magnitude, wash-in rate, and cumulative perfusion. CEUS-derived parameters, however, measure tissue perfusion rather than vessel number itself and may also be influenced by the vascular caliber, local blood flow, and microbubble concentration [10,11]. The observed associations should therefore be interpreted as a relationship between perfusion characteristics and local microvascularity rather than as a direct correspondence between CEUS measurements and vascular density.

In contrast, RT and TTP were significantly shorter in the perilesional zone than in reference liver parenchyma but were not significantly correlated with CD34-positive cell density. These parameters primarily describe the time required for contrast to enter the tissue and reach peak enhancement. In addition to local microcirculatory properties, they may be affected by bolus delivery, systemic circulation time, local vascular resistance, and the route of blood supply [10,11]. RT and TTP may therefore capture regional differences in perfusion timing without necessarily varying in parallel with local CD34-positive cell density. The different association patterns observed for enhancement- and wash-in-related parameters versus temporal parameters further indicate that individual TIC metrics characterize distinct, although partly overlapping, aspects of perilesional perfusion.

CD34-positive cell density, digitally quantified as the number of positive cells per unit tissue area, was used as a continuous histological surrogate of perilesional microvascularity. This metric differs from conventional microvessel density based on counting discrete vascular profiles. Although CD34 immunostaining and digital image analysis are commonly used to quantify endothelial structures [6,7], CD34 expression is not entirely specific to mature functional capillaries and may also occur in other stromal cell populations. Accordingly, the observed associations should be interpreted as relationships between CEUS-derived perfusion characteristics and CD34-positive microvascularity, rather than as direct evidence of microvessel number, functional perfused vessel density, vessel maturity, caliber, or patency.

Under the standardized image analysis protocol, quantitative CEUS measurements demonstrated good overall reproducibility. Full parameter- and ROI-specific ICC estimates with bootstrap 95% CIs are provided in Supplementary Table S3. ICC(A,2) estimates based on the mean of two observers were generally higher than the corresponding single-measurement ICC(A,1) estimates, indicating greater stability after averaging independent measurements and supporting the use of the mean of the two observers’ initial measurements in the primary analyses. Nevertheless, the precision of the ICC estimates varied across parameters and ROIs, with perilesional WiPI showing the widest interobserver ICC(A,1) CI; therefore, point estimates should be considered together with their CIs.

Bland–Altman analyses and SEM values complemented the ICC results by quantifying absolute measurement agreement for PE, WiR, WiPI, and WiAUC. Across the 16 observer- and ROI-specific comparisons, 90–100% of paired observations fell within the empirical 95% LoA; however, LoA widths and SEM% varied across parameters and measurement conditions (Supplementary Tables S4 and S5; Supplementary Figure S1). Taken together, the reproducibility analyses support the feasibility of quantitative CEUS under a standardized protocol while emphasizing the importance of consistent ROI placement and motion tracking.

Several limitations define the scope of these findings. First, the sample size was modest, and all assessments were performed at a single time point 10 weeks after inoculation. This design precluded evaluation of longitudinal changes and limited inference regarding disease activity, progression, and treatment response. Second, the standardized model in female SD rats produced a relatively homogeneous lesion phenotype. Although this improved experimental consistency, it limits generalizability across sexes, strains, disease stages, and the broader morphological spectrum of human HAE. Third, imaging–pathology correspondence was established at the target lesion and imaging plane level rather than by strict two- or three-dimensional registration. Histological fields were selected by one blinded pathologist within a morphologically defined inflammatory zone without independent review, leaving potential spatial mismatch and field-selection variability. Finally, CD34-positive cell density was the sole pathological vascular endpoint and does not capture vessel functionality or maturity, while CEUS measurements may also be affected by contrast agent delivery, systemic circulation, curve fitting, motion tracking, and manual ROI placement. Within these boundaries, the present study provides a reproducible quantitative framework for relating perilesional CEUS perfusion to CD34-positive microvascularity. Longitudinal studies and human validation with improved imaging–pathology registration and complementary vascular markers are needed to determine its translational utility.

## 5. Conclusions

In this rat model of HAE, quantitative CEUS characterized a distinct perilesional perfusion pattern. PE, WiR, WiPI, and WiAUC were positively correlated with CD34-positive cell density, supporting their potential as quantitative imaging indicators of perilesional microvascularity. These findings provide a basis for future longitudinal studies and clinical validation.

## Figures and Tables

**Table 1:** Quantitative CEUS parameters in the perilesional zone and reference liver parenchyma. Data are presented as mean ± standard deviation or median (interquartile range), as appropriate. Paired differences were calculated as perilesional zone minus reference liver values and are reported as mean differences (95% CI) for PE and RT or median differences (95% BCa bootstrap CI; 50,000 resamples) for TTP, WiR, WiPI, and WiAUC. FDR-adjusted *p*-values were calculated using the Benjamini–Hochberg procedure across the six comparisons.

Parameter	Perilesional Zone	Reference Liver Parenchyma	Raw *p*-Value	FDR-Adjusted *p*-Value	Paired Difference (95% CI)
PE (a.u.)	28,197.36 ± 7030.72	16,314.34 ± 3077.40	<0.001	<0.001	11,883.02 (8697.01–15,069.02)
RT (s)	13.01 ± 3.48	19.82 ± 4.37	<0.001	<0.001	−6.81 (−8.76 to −4.85)
TTP (s)	16.26 ± 4.25	21.94 ± 5.28	<0.001	<0.001	−4.59 (−7.04 to −3.13)
WiR (a.u.)	4069.44 (2744.70–5120.19)	1357.95 (1065.44–1603.97)	<0.001	<0.001	2467.08 (1689.78–3234.99)
WiPI (a.u.)	20,229.57 (16,286.73–22,889.84)	8284.60 (7733.29–9891.50)	<0.001	<0.001	10,180.54 (8153.88–13,403.12)
WiAUC (a.u.)	263,018.63 ± 89,548.90	172,188.46 ± 41,883.36	<0.001	<0.001	62,348.61 (37,006.40–125,441.99)

**Table 2:** Associations between CEUS parameters and CD34-positive cell density (cells/mm^2^). Spearman’s rank correlation was prespecified for the imaging pathology analysis. Marginal 95% CIs were estimated using 50,000 BCa animal-level bootstrap resamples. Raw *p*-values are reported numerically, and FDR-adjusted *p*-values were calculated across the six correlation tests using the Benjamini–Hochberg procedure.

Parameter	Spearman’s ρ	Raw *p*-Value	FDR-Adjusted *p*-Value	BCa 95% CI
PE	0.696	0.001	0.004	0.388–0.868
RT	−0.018	0.940	0.940	−0.480–0.475
TTP	−0.316	0.175	0.210	−0.718–0.247
WiR	0.525	0.018	0.026	0.141–0.785
WiPI	0.614	0.004	0.012	0.194–0.841
WiAUC	0.534	0.015	0.026	0.036–0.823

## Data Availability

The data generated and analyzed during this study are available from the corresponding author upon reasonable request.

## Supplementary Materials

The following supporting information can be downloaded at: https://www.mdpi.com/article/10.3390/pathogens15090987/s1, Figure S1: Bland–Altman plots for interobserver and intraobserver agreement of PE, WiR, WiPI, and WiAUC in the perilesional zone and reference liver parenchyma; Figure S2: Positive and negative controls for CD34 immunohistochemistry; Figure S3: Assessment of protoscolex viability using the eosin exclusion test; Table S1: leave-one-out sensitivity analyses of the associations between CEUS parameters and CD34-positive cell density; Table S2: Maximum lesion diameter associations with perilesional CEUS parameters; Table S3: Reproducibility of quantitative CEUS measurements by parameter and ROI; Table S4: Bland–Altman agreement analysis of quantitative CEUS measurements; Table S5: Standard error of measurement for quantitative CEUS parameters; File S1: Supplementary dataset; File S2: Supplementary statistical-analysis code.
