# Supplementary material for: Quantitative Contrast-Enhanced Ultrasound Characterization of Perilesional Perfusion and Its Association with CD34-Positive Cell Density in a Rat Model of Hepatic Alveolar Echinococcosis
Source: Pathogens. 2026 Sep 17;15(9):987. doi: 10.3390/pathogens15090987 (PMC13610811; doi:10.3390/pathogens15090987)
Supplement: Supplementary file 1 [file pathogens-15-00987-s001.zip › Supplementary Figure S1:Bland–Altman.pdf]

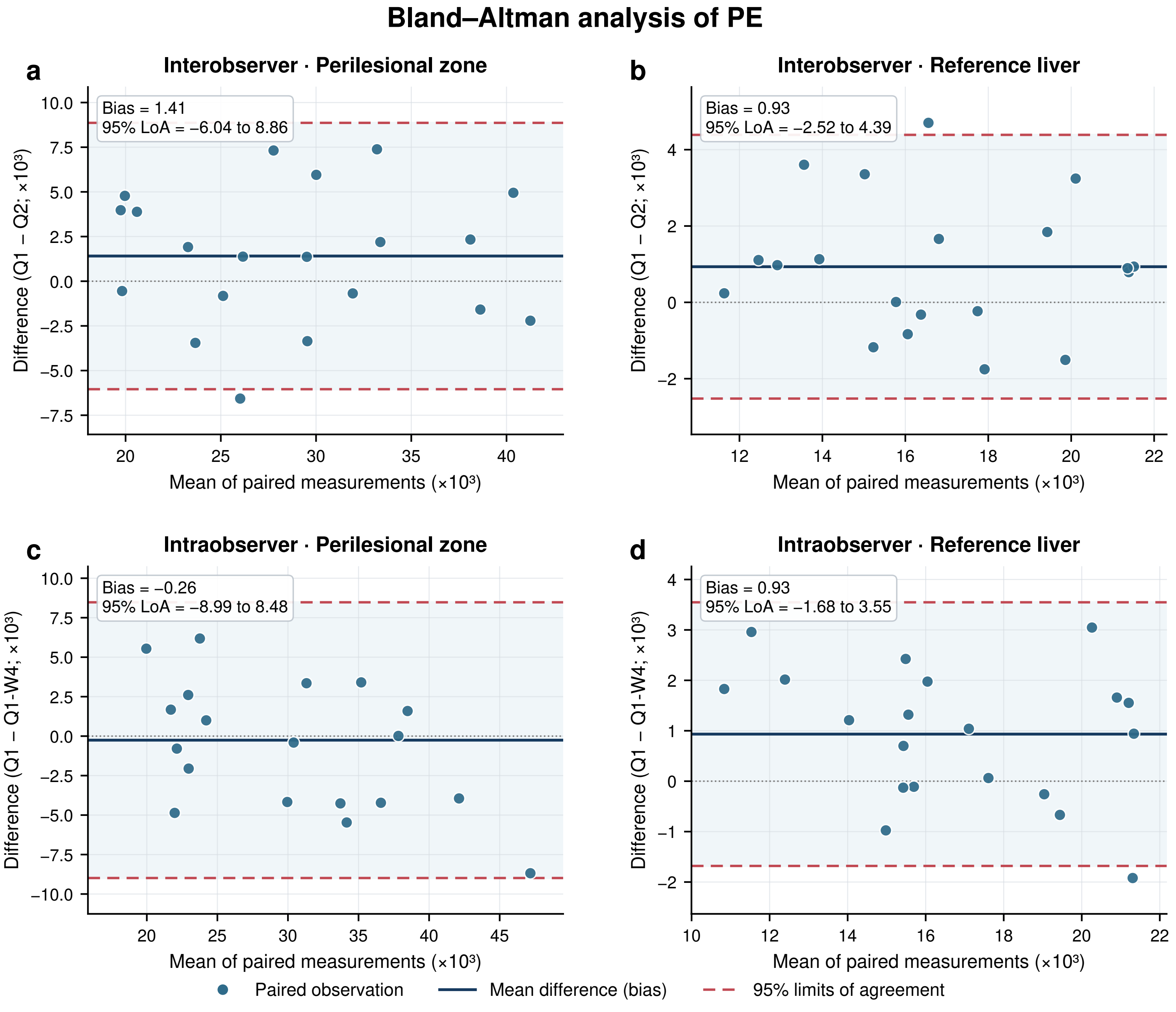

n = 20 paired measurements in each panel; values are shown in native software units scaled by 10³.

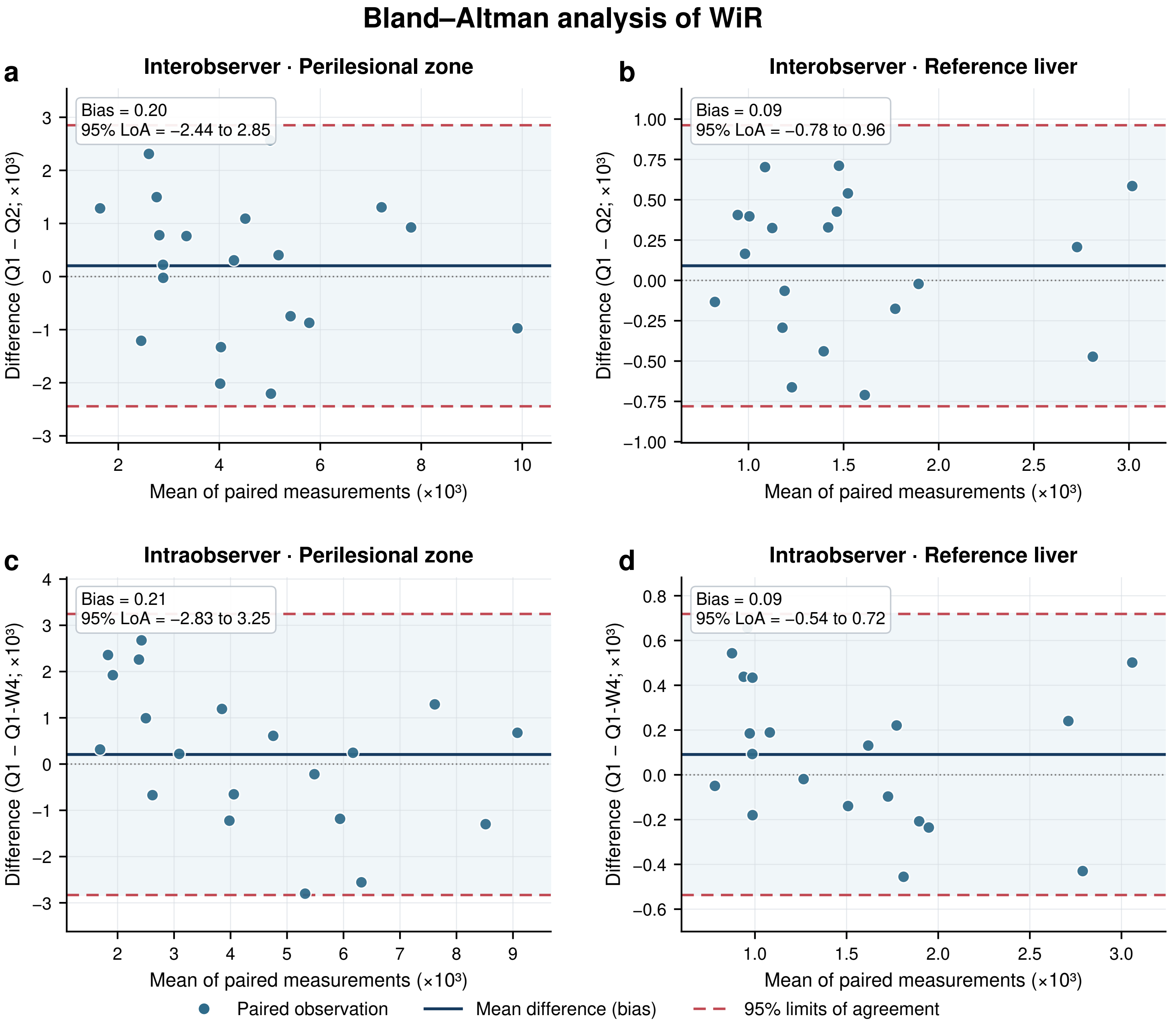

n = 20 paired measurements in each panel; values are shown in native software units scaled by 10³.

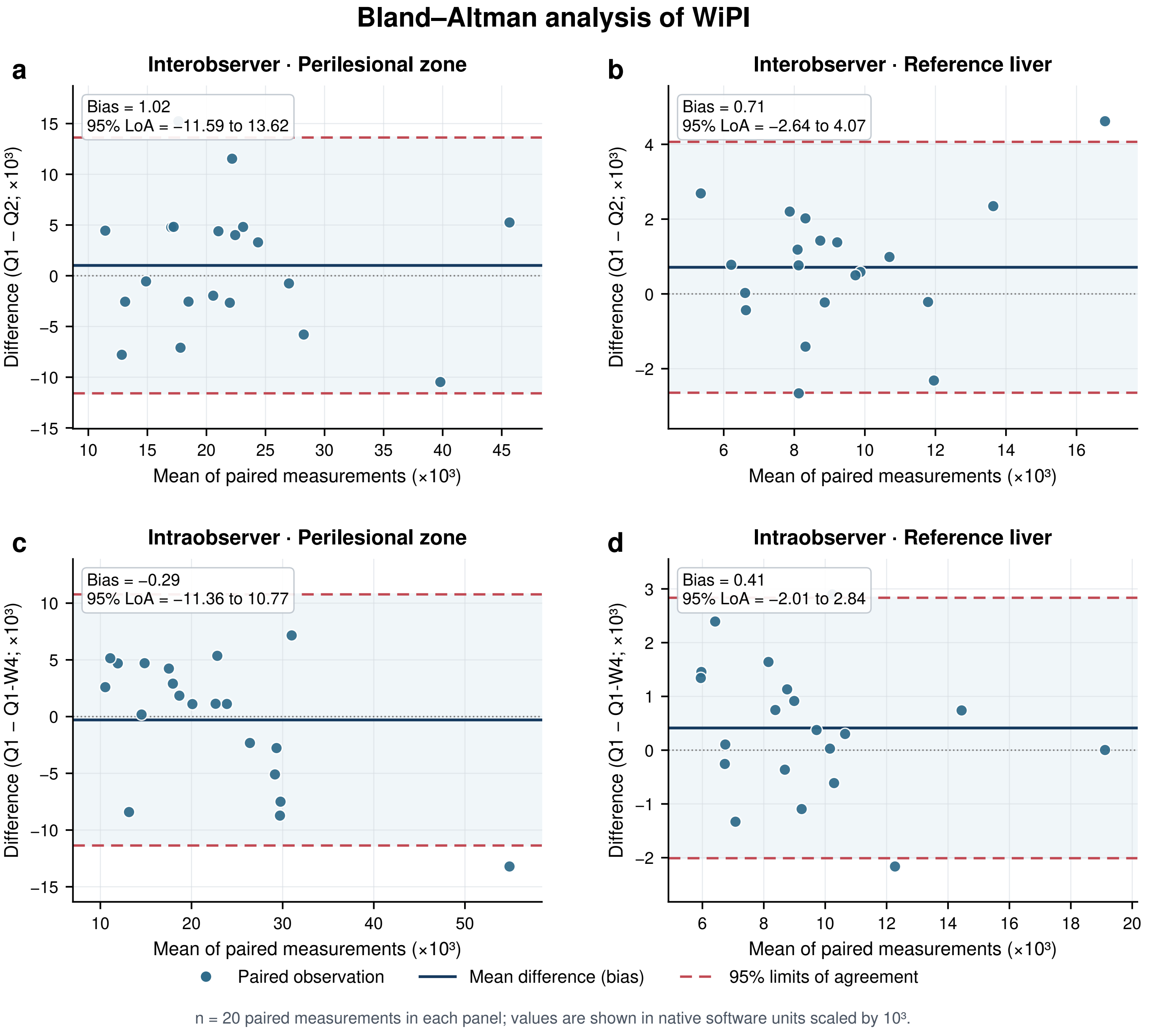
Bland–Altman analysis of WiPI
a
Interobserver · Perilesional zone
Bias = 1.02
95% LoA = −11.59 to 13.62
Difference (Q1 − Q2; ×10³)
Mean of paired measurements (×10³)
b
Interobserver · Reference liver
Bias = 0.71
95% LoA = −2.64 to 4.07
Difference (Q1 − Q2; ×10³)
Mean of paired measurements (×10³)
c
Intraobserver · Perilesional zone
Bias = −0.29
95% LoA = −11.36 to 10.77
Difference (Q1 − Q1-W4; ×10³)
Mean of paired measurements (×10³)
d
Intraobserver · Reference liver
Bias = 0.41
95% LoA = −2.01 to 2.84
Difference (Q1 − Q1-W4; ×10³)
Mean of paired measurements (×10³)
Paired observation
Mean difference (bias)
95% limits of agreement
n = 20 paired measurements in each panel; values are shown in native software units scaled by 10³.

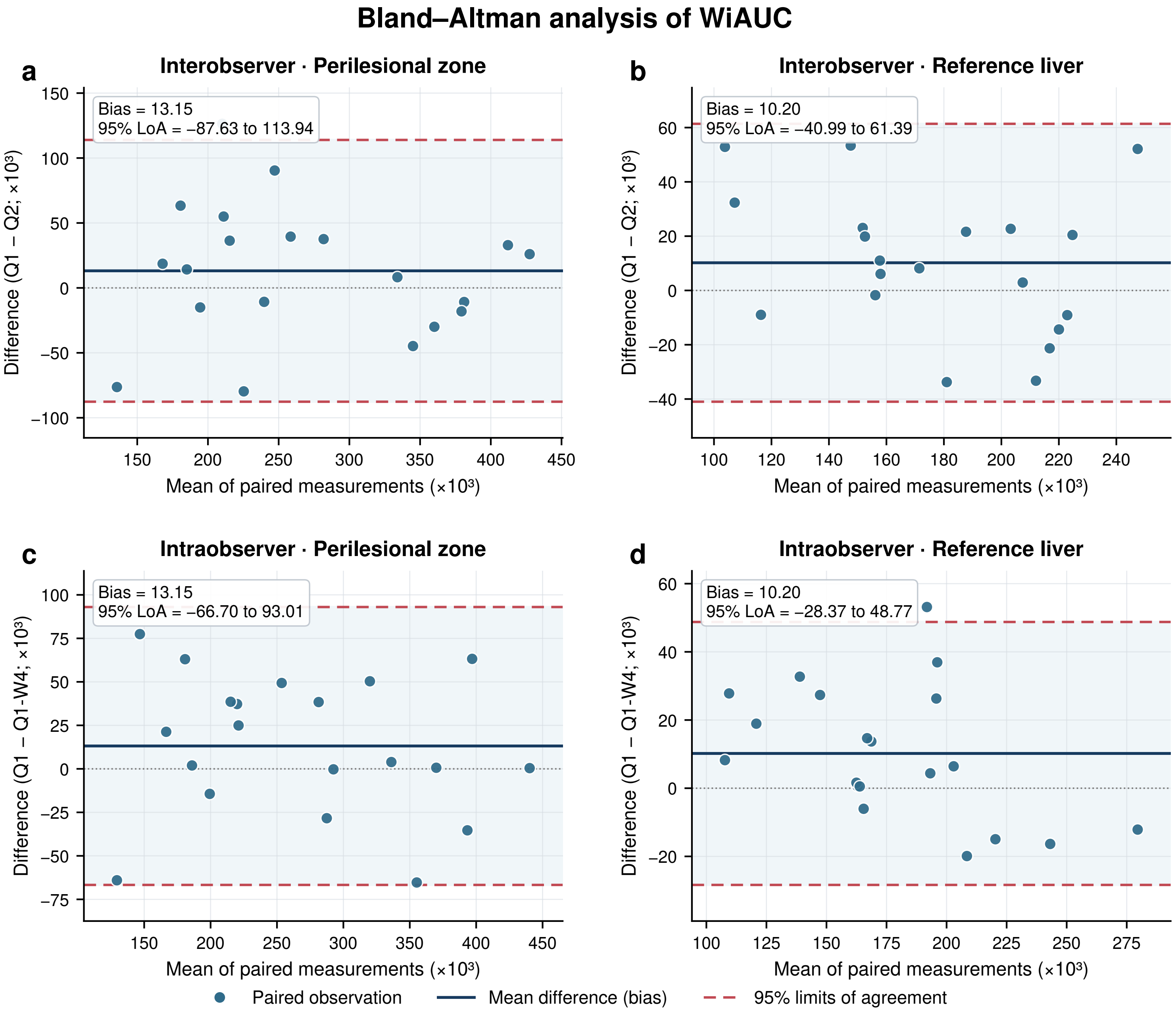

n = 20 paired measurements in each panel; values are shown in native software units scaled by 10³.
