# Supplementary material for: Quantitative Contrast-Enhanced Ultrasound Characterization of Perilesional Perfusion and Its Association with CD34-Positive Cell Density in a Rat Model of Hepatic Alveolar Echinococcosis
Source: Pathogens. 2026 Sep 17;15(9):987. doi: 10.3390/pathogens15090987 (PMC13610811; doi:10.3390/pathogens15090987)
Supplement: Supplementary file 1 [file pathogens-15-00987-s001.zip › Supplementary Tables.pdf]

Supplementary Table S1. leave-one-out sensitivity analyses of the associations between CEUS parameters and CD34-positive cell density.

Parameter	Leave-one-out ρ range	FDR-significant iterations
PE	0.651 – 0.749	20/20
RT	−0.188 – 0.091	0/20
TTP	−0.470 – 0.230	0/20
WiR	0.463 – 0.591	15/20
WiPI	0.560 – 0.698	20/20
WiAUC	0.456 – 0.665	17/20

Supplementary Table S2. maximum lesion diameter associations with perilesional CEUS parameters.

Parameter	Spearman ρ	Raw P	FDR-adjusted P
PE	0.105	0.659	0.910
RT	0.098	0.682	0.910
TTP	0.134	0.574	0.910
WiR	0.027	0.910	0.910
WiPI	-0.150	0.527	0.910
WiAUC	-0.068	0.777	0.910

Supplementary Table S3. Reproducibility of quantitative CEUS measurements by parameter and ROI.

Parameter	ROI	Interobserver ICC(A,1) (95% CI)	Interobserver ICC(A,2) (95% CI)	Intraobserver ICC(A,1) (95% CI)
PE	Perilesional zone	0.873 (0.713–0.939)	0.932 (0.832–0.969)	0.858 (0.773–0.902)
PE	Reference liver	0.854 (0.682–0.933)	0.921 (0.811–0.965)	0.918 (0.852–0.953)
RT	Perilesional zone	0.864 (0.717–0.931)	0.927 (0.835–0.964)	0.932 (0.843–0.967)
RT	Reference liver	0.844 (0.709–0.914)	0.916 (0.830–0.955)	0.922 (0.793–0.967)
TTP	Perilesional zone	0.834 (0.611–0.928)	0.910 (0.759–0.963)	0.817 (0.615–0.886)
TTP	Reference liver	0.824 (0.639–0.907)	0.903 (0.779–0.951)	0.931 (0.853–0.967)
WiR	Perilesional zone	0.813 (0.491–0.914)	0.897 (0.659–0.955)	0.801 (0.596–0.899)
WiR	Reference liver	0.792 (0.359–0.889)	0.884 (0.528–0.941)	0.898 (0.766–0.940)
WiPI	Perilesional zone	0.759 (0.256–0.894)	0.863 (0.408–0.944)	0.862 (0.705–0.906)
WiPI	Reference liver	0.822 (0.632–0.883)	0.902 (0.774–0.938)	0.929 (0.727–0.976)
WiAUC	Perilesional zone	0.854 (0.687–0.933)	0.921 (0.815–0.965)	0.912 (0.846–0.950)
WiAUC	Reference liver	0.834 (0.702–0.902)	0.910 (0.825–0.949)	0.905 (0.767–0.954)

Supplementary Table S4. Bland – Altman agreement analysis of quantitative CEUS measurements.

Parameter	ROI	Comparison	Mean bias	SD of paired differences	95% LoA
PE	Perilesional zone	Interobserver	1409.87	3802.85	−6043.72 - 8863.46
PE	Perilesional zone	Intraobserver	−255.53	4454.74	−8986.82 - 8475.76
PE	Reference liver	Interobserver	933.44	1762.45	−2520.96 - 4387.84
PE	Reference liver	Intraobserver	933.44	1333.91	−1681.03 - 3547.91
WiR	Perilesional zone	Interobserver	203.47	1350.49	−2443.48 - 2850.42
WiR	Perilesional zone	Intraobserver	207.14	1550.16	−2831.16 - 3245.45
WiR	Reference liver	Interobserver	90.87	444.47	−780.29 - 962.03
WiR	Reference liver	Intraobserver	90.88	320.28	−536.86 - 718.63
WiPI	Perilesional zone	Interobserver	1016.45	6432.86	−11591.96 - 13624.86
WiPI	Perilesional zone	Intraobserver	−294.23	5646.75	−11361.87 - 10773.41
WiPI	Reference liver	Interobserver	711.66	1711.53	−2642.93 - 4066.26
WiPI	Reference liver	Intraobserver	412.58	1235.93	−2009.84 - 2835.01
WiAUC	Perilesional zone	Interobserver	13150.93	51421.06	−87634.35 - 113936.21
WiAUC	Perilesional zone	Intraobserver	13150.93	40742.63	−66704.62 - 93006.48
WiAUC	Reference liver	Interobserver	10199.11	26116.06	−40988.36 - 61386.58
WiAUC	Reference liver	Intraobserver	10199.11	19676.68	−28367.18 - 48765.40

Supplementary Table S5. Standard error of measurement for quantitative CEUS parameters.

Parameter	ROI	Interobserver SEM	Interobserver SEM (%)	Intraobserver SEM	Intraobserver SEM (%)
PE	Perilesional zone	2787.22	9.64	3085.39	10.38
PE	Reference liver	1358.93	8.10	1112.47	6.63
WiR	Perilesional zone	944.34	21.09	1082.01	24.17
WiR	Reference liver	313.28	20.43	229.54	14.97
WiPI	Perilesional zone	4505.58	20.63	3910.38	17.38
WiPI	Reference liver	1268.35	13.71	898.33	9.56
WiAUC	Perilesional zone	36595.75	13.57	29504.30	10.94
WiAUC	Reference liver	19214.85	10.84	15196.22	8.57
